# COVID-19-Related Pulmonary Embolism: Incidence, Characteristics, and Risk Factors

**DOI:** 10.7759/cureus.19738

**Published:** 2021-11-19

**Authors:** Ziad M Bukhari, Mohammed S Alqarni, Abdulkarim W Abukhodair, Ali S Alzahrani, Abdulmalek Alzahrani, Hetaf Alsrhani, Farah Alasadi, Abdullah M Alotaibi, Mohammed Althobaiti

**Affiliations:** 1 Medicine, King Abdullah International Medical Research Center (KAIMRC), Jeddah, SAU; 2 Medicine, King Saud Bin Abdulaziz University for Health Sciences, Jeddah, SAU; 3 Department of Medical Imaging, King Abdulaziz Medical City National Guard Hospital, Jeddah, SAU; 4 Department of Radiology, King Abdulaziz Medical City National Guard Hospital, Jeddah, SAU; 5 Radiology, King Abdullah International Medical Research Center (KAIMRC), Jeddah, SAU

**Keywords:** covid-19, ct, risk factors, prevalence, pulmonary embolism

## Abstract

Introduction: The 2020 world pandemic caused by the novel coronavirus severe acute respiratory syndrome coronavirus 2 (SARS-CoV-2) was initially reported in December 2019 in Wuhan, China, which has since then spread globally. Several studies on patients with coronavirus disease 2019 (COVID-19) describe a high risk of pulmonary embolism (PE). The majority of PEs in patients with COVID-19 were in the segmental arteries. Therefore, this study aims to determine the rate of PE in patients with COVID-19 at King Abdulaziz Medical City in Jeddah, Saudi Arabia. Other risk factors of PE were taken into consideration.

Patients and Methods: This study is a single-center, retrospective, cross-sectional study that used a non-probability consecutive sampling technique to select the patients. The local institutional review boards approved the study protocol. Overall, 91 consecutive patients who were older than 18 years of age and who had a computerized tomography (CT) pulmonary angiography were included in this study.

Results: Ninety-one patients met the inclusion and exclusion criteria, of whom 46 (50.5%) were females and 45 (49.5%) were males. The study population’s age ranged from 19 to 87 with a mean age of 59 ± 15 years. PE was documented in 11 patients (12.1%). Seventy-three patients underwent CT scan angiography during COVID-19 manifestation, while 18 patients had it after recovering from COVID-19. Out of the 11 patients with PE, eight were diagnosed with PE while being COVID-19 positive, and three were diagnosed with PE after recovery from COVID-19.

Conclusion: Several potential clinical implications can be concluded for this study. Firstly, effective evaluation of the risk of PE in patients with COVID-19 is based on clinical findings such as chest pain, hemoptysis, lower limb edema, and, most significantly, shortness of breath. Secondly, measuring D-dimer remains an effective test for ruling out PE in patients with COVID-19 as in patients without COVID-19.

## Introduction

The novel coronavirus severe acute respiratory syndrome coronavirus 2 (SARS-CoV-2), also known as coronavirus disease 2019 (COVID-19), originated in December 2019 in Wuhan, China. It has ever since rapidly spread worldwide, causing morbidity and mortality in its way. In March 2020, the World Health Organization (WHO) declared the COVID-19 outbreak a pandemic [1,2].

Patients with COVID-19 primarily developed respiratory tract infections, and in critically ill patients, it leads to respiratory failure and multiple organ failure [3]. Acute inflammation caused by severe infection or sepsis can alter the coagulation status [4]. Almost 20% of patients with coronavirus disease 2019 (COVID‐19) developed severe abnormalities in the coagulation status, mainly manifested by hypercoagulable state [3-5]. Higher levels of plasma D-dimer on admission were documented in COVID-19-related deaths compared with patients who survived [6-12]. In addition to the coagulation marker abnormalities, both venous and arterial thrombosis have been associated with COVID-19 infection [13,14]. An extremely high cumulative incidence of thrombotic complications was found in critically ill patients with COVID-19 pneumonia [13].

Several studies on patients with COVID-19 describe a high risk of pulmonary embolism (PE) [15-22]. The majority of PEs in patients with COVID-19 were in the segmental arteries [21,23,24]. Increased frequency of thromboembolic events, including pulmonary vasculature thrombosis, was confirmed by autopsy among deceased patients due to COVID-19 infection [23-27].

The early recognition of PE in patients with COVID-19 is essential to ensure proper management and a better prognosis [28]. The results of recent studies devoted to assessing coronavirus-related pulmonary embolism have been variable. Therefore, this study aims to determine the rate of PE in patients with COVID-19 at King Abdulaziz Medical City in Jeddah, Saudi Arabia. Other risk factors of PE were taken into consideration.

## Materials and methods

This study is a single-center, retrospective, cross-sectional study that used a non-probability consecutive sampling technique to select the patients. The local institutional review board of King Abdullah International Medical Research Center issued approval JED-21-427780-104397. Ninety-one consecutive patients who were older than 18 years of age and who had a computerized tomography (CT) pulmonary angiography from March 1, 2020, to March 13, 2021, at King Abdulaziz Medical City (Jeddah, Saudi Arabia) were identified through the picture archive and communication system for all patients with COVID-19. We excluded patients who had COVID-19 and did not undergo a CT study. 

A table with the following demographics was constructed: age, gender, body mass index (BMI), past medical history, D-dimer, presence of PE, type of PE, clinical symptoms, date of diagnosis, vital signs on CT scan, and mortality. The data were collected from an electronic database (Best care and picture archive and communication system), and two experienced radiologists reviewed all the CT studies.

CT scans were performed using three scanners (GE Lightspeed VCT 64 Slice CT scanner (GE Healthcare, Chicago, Illinois, United States), GE HD 64 Slice CT Scanner (GE Healthcare, Chicago, Illinois, United States), and Dual-Source 128 Slice CT SOMATOM Definition Flash CT Scanner (Siemens, Forchheim, Germany)). Axial images were obtained with a slice thickness of 0.6 mm, with sagittal and coronal reformats of slice thickness of 3 mm. All studies were reviewed and verified by an experienced consultant radiologist.

For the analysis, categorical variables are presented as frequencies and percentages and continuous variables as standard deviations or medians; interquartile ranges were used when the distributions were skewed. A p-value < 0.05 was considered significant. All results were analyzed using IBM SPSS version 23.

## Results

Ninety-one unvaccinated patients met the inclusion and exclusion criteria, of whom 46 (50.5%) were females and 45 (49.5%) were males. The study population’s age ranged from 19 to 87 with a mean age of 59 ± 15 years. PE was documented in 11 patients (12.1%). Seventy-three patients underwent CT scan angiography during COVID-19 manifestation, while 18 patients had it after recovering from COVID-19. Out of the 11 patients with PE, eight were diagnosed with PE while being COVID-19 positive, and three were diagnosed with PE after recovery from COVID-19. The demographics and risk factors for patients with and without PE are presented in Table 1.

**Table 1:** Demographics and risk factors for the PE and non-PE groups. PE: pulmonary embolism; DVT: deep vein thrombosis; BMI: body mass index. *Chi-square test or Fisher’s exact test was used.

Variables	Total sample (n = 91)	Patients with PE (n = 11)	Patients without PE (n = 80)	p-value*
Gender				0.7
Male	45	5 (11.1%)	40 (88.9%)	
Female	38	6 (13%)	40 (87%)	
BMI				0.8
Underweight	3	-	3 (100%)	
Normal	10	1 (10%)	9 (90%)	
Overweight	28	3 (10.7%)	25 (89.3%)	
Obese	50	7 (14%)	43 (86%)	
Dyslipidemia				0.4
Yes	40	6 (15%)	34 (85%)	
No	50	5 (10%)	45 (90%)	
Smoking				0.550
Yes	11	2 (18.2%)	9 (81.8%)	
No	80	9 (11.2%)	71 (88.8%)	
Immobility for three days				0.141
Yes	12	3 (25%)	9 (75%)	
No	79	8 (10.1%)	71 (89.9%)	
History of DVT or PE				0.07
Yes	4	2 (50%)	2 (50%)	
No	87	9 (10.3%)	78 (81.3%)	

Recent surgery within the previous four weeks (n = 2), heart failure (n = 6), atrial fibrillation (n = 2), pregnancy (n = 2), estrogen use (n= 2), and lower limb injury (n = 1) were also recorded in only non-PE patients. At the time of the CT scan angiography, the study population presented a mean heart rate of 97 ± 17 beats/minute, respiratory rate of 26 ± 6 breaths/minute, oxygen saturation of 93% ± 7%, systolic blood pressure of 130 ± 18 mmHg, and temperature of 37.2°C ± 0.7°C.

D-dimer concentration was measured in 74 patients, and it was high in all 11 patients with PE. For the non-PE patients, D-dimer concentration was high in 52 patients and normal in 11 patients. Based on the data, D-dimer had a sensitivity of 100% and a specificity of 17.5%.

Out of the 91 patients, 60 suffered from clinical signs and symptoms of PE. These symptoms included shortness of breath (n = 36), cough (n = 35), chest pain (n = 7), lower limb edema (n = 5), and hemoptysis (n = 5). There was no difference between the PE and non-PE groups in the signs and symptoms, except for shortness of breath, for which the patients with PE had a higher rate of incidence (25%) than patients without PE (3.6%) (p-value = 0.006).

The study population included 19 patients with malignancy. Only one case of PE was associated with malignancy (lung cancer). All the types of malignancy documented can be found in Table 2.

**Table 2:** Various types of malignancy detected in the study population.

Type of malignancy	n (%)
Lymphoma	6 (6.6%)
Breast cancer	3 (3.3%)
Colorectal cancer	3 (3.3%)
Nasopharyngeal cancer	2 (2.2%)
Lung cancer	1 (1.1%)
Leukemia	1 (1.1%)
Bladder cancer	1 (1.1%)
Renal cancer	1 (1.1%)
Maxillary angiosarcoma	1 (1.8)
No malignancy	72 (79.1%)

There were eight deceased patients (mortality rate: 8.8%), one of whom suffered from PE. There was no statistical difference in mortality between the PE and non-PE groups. Various lung alterations were detected by CT imaging in the total sample (Table 3).

**Table 3:** Lung alterations detected in the study population. COVID: coronavirus disease.

Lung changes	n (%)
Post-COVID sequelae including fibrosis and post-COVID organizing pneumonia	20 (22%)
COVID pneumonia changes	58 (63.7%)
Infraction	3 (3.3%)
Metastasis	2 (2.2%)
Others	7 (7.7%)
No lung alterations	1 (1.1%)

The patients with PE (n = 11) presented various anatomical locations of the embolus. Also, some patients presented PE in more than one site, as shown in Table 4.

**Table 4:** Anatomical locations of the emboli in 11 patients with PE. PE: pulmonary embolism.

PE location	n (%)
Segmental PE	7 (7.7%)
Sub-segmental PE	6 (6.6%)
Major PE	2 (2.2%)
Lobar PE	1 (1.1%)

## Discussion

This study assessed the incidence of pulmonary embolism among patients with COVID-19 and post-COVID-19 pneumonia who underwent CT pulmonary angiography because of clinical suspicion of PE. Out of 91 patients diagnosed with COVID-19, 11 developed PE (12.1%). This incidence is similar to that reported in Riyadh, Saudi Arabia (11.6%) [29]. Many other studies showed a higher incidence. For example, in a study conducted in France with a sample size of 106 patients with COVID-19, the incidence of acute PE was 30% [30]. Another research showed an incidence of 31% for venous and arterial thrombotic events in a sample of 184 intensive care unit patients [10]. In our study, patients presented PE either when actively infected with COVID-19 (n = 8) or in the postinfection period (n = 3). The principal pulmonary embolisms were segmental and sub-segmental, with only two major PE and one lobar PE case. These results supported a few publications that showed predominantly segmental PE [21,23,24]. This could result in a limited impact on hemodynamic stability and prognosis. In our study, out of the 11 patients with COVID-19 with PE, one died.

Many other risk factors of pulmonary embolism are known, including recent surgery, acute or chronic medical illness, malignancies, hormonal-related factors, known thrombophilia, BMI > 30, prior history of PE or deep vein thrombosis, and prolonged immobilization or travel [31]. We included almost all of the aforementioned risk factors in our analysis. Recent surgery, heart failure, atrial fibrillation, pregnancy, estrogen use, and lower limb injury were found in part of our sample; however, these patients did not develop PE. On the other hand, out of the 11 patients with PE, six had dyslipidemia, two were smokers, three were immobile for the last three days, seven suffered from obesity, and two had a prior history of deep vein thrombosis or PE. These factors may have been individual or contributing factors to developing PE after infection with COVID-19.

Malignancy was the main concern regarding interfering with the risk of developing PE since the study was conducted in a tertiary center. Nonetheless, out of the 11 patients with PE, only one was diagnosed with lung cancer. Out of the 11 patients with PE, three had secondary complications of pulmonary infarction, and one developed right heart strain. The overall mortality rate (in both patients with and without PE) was 8.8%. There was only one mortality case in the patients with PE. 

Most patients with COVID-19 face difficulties in taking a deep breath and holding it during CT pulmonary angiogram acquisition, mainly due to shortness of breath and chest pain. This compromises the detection of peripheral PE [15-17]. Although no studies were undetermined considering the diagnosis of PE, we must consider that this might have led to an underestimation of sub-segmental PE due to technical issues or underlying lung disease. Both PE and non-PE COVID-19 groups presented a wide range of symptoms, including dyspnea, cough, chest pain, lower limb edema, and hemoptysis. Patients who complained of shortness of breath showed a higher rate of PE (25%) compared with those who did not present it (3.6%).

D-dimer levels increase because of coagulation [32]. However, other causes of high D-dimer concentrations include inflammation, infection, trauma, post-surgery complications, coronary artery disease, and malignancy [32]. Therefore, D-dimer levels are highly sensitive to various conditions, with a lower specificity in diagnosing PE and other thrombotic diseases [33]. D-dimer measurements were available in 74 patients of the total sample. D-dimer levels were elevated in all patients with PE and 52 patients without PE, and they were normal in 11 patients without PE. The calculated sensitivity and specificity were 100% and 17.5%, respectively. In a study performed in Wuhan, D-dimer sensitivity was lower (85%) than the one presented here, but specificity had a higher value (88.5%) [34]. Significantly elevated D-dimer levels are associated with severe disease and poor prognosis [9,35,36]. COVID-related pneumonia alterations were found in 63.7% of the study population, while post-COVID-19 fibrotic changes and organizing pneumonia were found in 22% [37].

## Conclusions

Several potential clinical implications can be concluded for this study. Firstly, effective evaluation of the risk of PE in patients with COVID-19 is based on clinical findings such as chest pain, hemoptysis, lower limb edema, and, most significantly, shortness of breath. Secondly, measuring D-dimer remains an effective test for ruling out PE in patients with COVID-19 as in patients without COVID-19. Finally, assessing patients using CT is essential in diagnosing PE and improving patient outcomes.
